# Identifying Blunt Force Traumatic Injury on Thermally Altered Remains: A Pilot Study Using *Sus scrofa*

**DOI:** 10.3390/biology11010087

**Published:** 2022-01-06

**Authors:** Kamryn Keys, Ann H. Ross

**Affiliations:** Department of Biological Sciences, North Carolina State University, Raleigh, NC 27695, USA; kkeys@ncsu.edu

**Keywords:** forensic anthropology, forensic science, blunt force trauma, thermal alteration, thermal fractures

## Abstract

**Simple Summary:**

Human remains are often burned in an effort to conceal the identity of the victim and/or obscure traumatic injuries related to the death event. Thermal exposure can produce artifacts resembling trauma and disguise preexisting trauma. However, there is a paucity of experimental studies with varied results addressing the differentiation of thermally induced artifacts from traumatic signatures. To address this gap in the literature, we conducted a small-scale study using domestic pigs as correlates to test the impact of thermal alteration on blunt force trauma to the cranium. Two tools (e.g., hammer and crowbar) were utilized to manually inflict injuries on the human analogs before controlled burning in an outdoor environment. The results of this experiment demonstrated that the most diagnostic variable to differentiate thermally induced alternations from blunt force fractures was fracture pattern.

**Abstract:**

In forensic scenarios involving homicide, human remains are often exposed to fire as a means of disposal and/or obscuring identity. Burning human remains can result in the concealment of traumatic injury, the creation of artifacts resembling injury, or the destruction of preexisting trauma. Since fire exposure can greatly influence trauma preservation, methods to differentiate trauma signatures from burning artifacts are necessary to conduct forensic analyses. Specifically, in the field of forensic anthropology, criteria to distinguish trauma from fire signatures on bone is inconsistent and sparse. This study aims to supplement current forensic anthropological literature by identifying criteria found to be the most diagnostic of fire damage or blunt force trauma. Using the skulls of 11 adult pigs (*Sus scrofa*), blunt force trauma was manually produced using a crowbar and flat-faced hammer. Three specimens received no impacts and were utilized as controls. All skulls were relocated to an outdoor, open-air fire where they were burned until a calcined state was achieved across all samples. Results from this experiment found that blunt force trauma signatures remained after burning and were identifiable in all samples where reassociation of fragments was possible. This study concludes that distinct patterns attributed to thermal fractures and blunt force fractures are identifiable, allowing for diagnostic criteria to be narrowed down for future analyses.

## 1. Introduction

Trauma interpretation is arguably one of the most valuable services a forensic anthropologist can perform to assist criminal investigative proceedings. This is evidenced by the consistent theme of trauma-focused research in the forensic anthropology literature, spanning several decades [1,2,3,4,5,6,7,8,9,10,11,12]. Special interest is, in part, due to the fact that biomechanical signatures of skeletal trauma are not fully understood [13,14,15,16]. As such, considerable research replicating traumatic force has been produced to document the resultant characteristics seen on bone [1,2,3,4,5,6,7,8,9,10,11,13]. Efforts to identify the source of trauma are only half of the assessment, as it is imperative for the timing of the injury to be established as well. To interpret injury timing, characteristics of the defect are noted concerning the reaction of the immediate surrounding bone [8,9,13,14,15,16]. Following visual observation of trauma, it is assigned to an ante-, peri-, or postmortem temporal context [3,4,8]. By establishing the timing of the defect, the anthropologist can provide insight into whether the injury potentially contributed to the death event [3,4,8,13,14,15,16].

Although the characteristics of blunt force impact are the focus of many studies [1,2,3,4,6,7,8,11,13,16], there is still much ambiguity surrounding trauma assessment. Major issues encountered when interpreting skeletal trauma include the influence of the deposition environment, endogenous and exogenous taphonomic processes, postmortem disturbance from scavengers, or relocation to secondary deposition sites [17,18,19,20,21,22]. All of these variables introduce the potential for trauma alterations that must be accounted for during skeletal analysis [17,18,19,20,21,22]. Although the variables influencing trauma interpretation differ from case to case, and even across elements of the same skeleton, the laws of bone biomechanics that guide these analyses stay constant [6,13,14,15,16]. The main consideration is that wet or living bone will respond to slow loading force (e.g., blunt force) by first absorbing the force through plastic deformation until the force overloads the bone causing it to fail (e.g., break) [6,11,12,13,14]. Plastic deformation is expressed in the bone as crushing of the cortical layer into the internal cancellous region, as the bone slowly absorbs force without exceeding its yield strength. Per contra, when a bone is exposed to rapid force, such as is seen with gunshot trauma, it will react as a more brittle material and fracture with little to no associated plastic deformation [6,7,8,13,14,15,16]. This brittle reaction is also characteristic of postmortem dry bone breakage [17,18,19,20,21,22,23,24,25,26]. Both plastic deformation and complete bone failure leave distinct signatures on the bone when observed both macro- and microscopically [6,7,8,17,18,19,20,21,22,23,24,25,26]. There is general agreement that if the biomechanics of bone’s reaction to force remain as a constant variable, then interpreting the timing of traumatic injury should be possible despite postmortem taphonomic events and alterations [6,7,8,17,18,19,20,21,22,23].

Although the structural reactions of traumatized bone are well understood, post-depositional events can complicate interpretation. Taphonomic processes can introduce secondary fractures, alter fracture margins, or conceal impact sites [6,24,25,26]. Due to this, trauma signatures are addressed in variable depositional environments [1,2,3,4,5,6,7,8,9,10,11,15,16,17,18,19,20,21,22,23,24,25,26,27,28] to identify criteria that can be informative for trauma identification in specific contexts. However, few studies have addressed the influence of thermal alteration on blunt force trauma, specifically on the cranium. In forensic contexts, it is not uncommon for decedents to be disposed of by means of fire, as perpetrators of a crime often correlate the idea of a quick coverup with burning the body until only ash remains [20,21,24,25,26,27,28]. However, bodies exposed to fire burn slowly and are often recovered with intact skeletal or fleshed elements remaining [21,24,25,26,27,28]. When bodies are exposed to fire for a significant time, heat will alter the bone by degrading its organic components, leaving only the mineral structure [6,23,24,25,26,27,28]. The organic components, which are quickly dehydrated and destroyed from thermal modification, are what allow plastic deformation in living bone [6,12,13,14,15]. Therefore, thermal fractures express features similar to bone impacted by rapid force [6,17,18,19,20,21,22,25,26,27,28]. Further investigation is needed to understand the modifications caused by thermal exposure to perimortem trauma, as conclusions from the existing literature are unclear, inconsistent, and without validation [1,3,4,5,6,7]. Research derived for applications in forensic contexts is unique in its necessity for the method to pass the rigors required in legal proceedings [29,30,31,32]. All methods applied in forensic testing must be guided by strict sets of procedures and criteria. Thus, this research aims to identify characteristics that are indicative of fracture origin in thermally altered remains. Specifically, this paper highlights the characteristics of thermal and mechanically derived fractures of the cranium using *Sus scrofa* analogs, this being one of the most commonly traumatized regions in forensic contexts.

## 2. Materials and Methods

**Figure 1:**
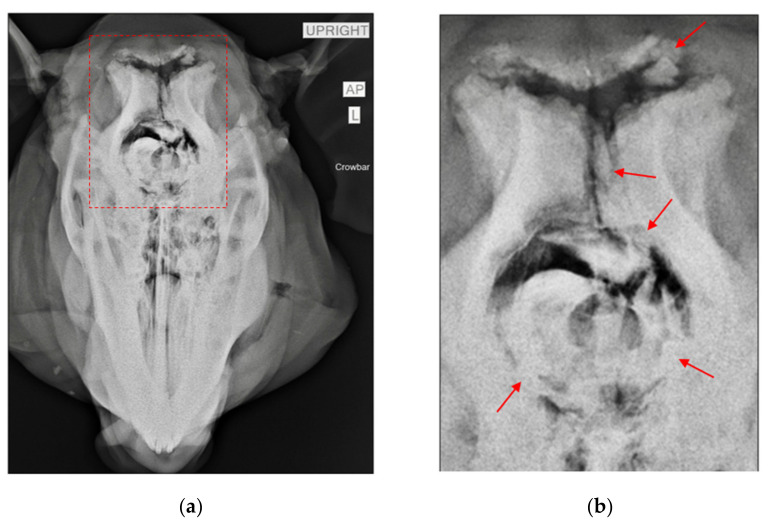
Radiographic image of sample CBH1. (**a**) Pre-burn radiograph of a specimen after manual trauma with a crowbar. Red dotted lines denote the area containing blunt force trauma. (**b**) Close-up image of the area within the red rectangle. Red arrows point to areas of incomplete fractures as a result of blunt force trauma, with associated fragments still attached.

This study used 11 adult pigs as proxies for human remains, due to an established similarity in tissue thickness and structure between humans and pigs [2,3,11]. The pigs were procured from a local pork center and were humanely euthanized the morning of the experiment with a blank bullet (following NC laws for humane slaughter) and shipped via cooling container to the pick-up site, where they were then transferred to the forensic laboratory. The samples consisted of skulls containing the complete cranium and mandible, and all were disarticulated from the axial skeleton prior to experimentation. No soft tissue was removed before experimentation, as removing tissue before blunt force trauma would not be consistent with an actual forensic event [24]. Of the 11 samples, 3 of the specimens were used as non-traumatized controls but were still subjected to burning. The remaining eight samples were divided into two groups, each containing four specimens (Table 1). One group was manually struck with a rounded crowbar and the other a flat-faced hammer. Two types of tools were used due to the different surface areas. Two samples of each group were traumatized with the head lying supine and the other two with the head positioned in the horizontal plane. This positioning allowed the recreation of forensic scenarios where a decedent is struck standing up (horizontal) or fallen (supine) with a buttressed surface creating secondary fractures opposite the initial impacts. Each specimen was struck on the frontal, zygomatic, parietal, and nasal bones. Samples were traumatized several times until fractures could be manually felt and then radiographed to document perimortem fracture patterns (Figure 1). Following radiographic documentation, the samples were taken to the burn site.

**Figure 2:**
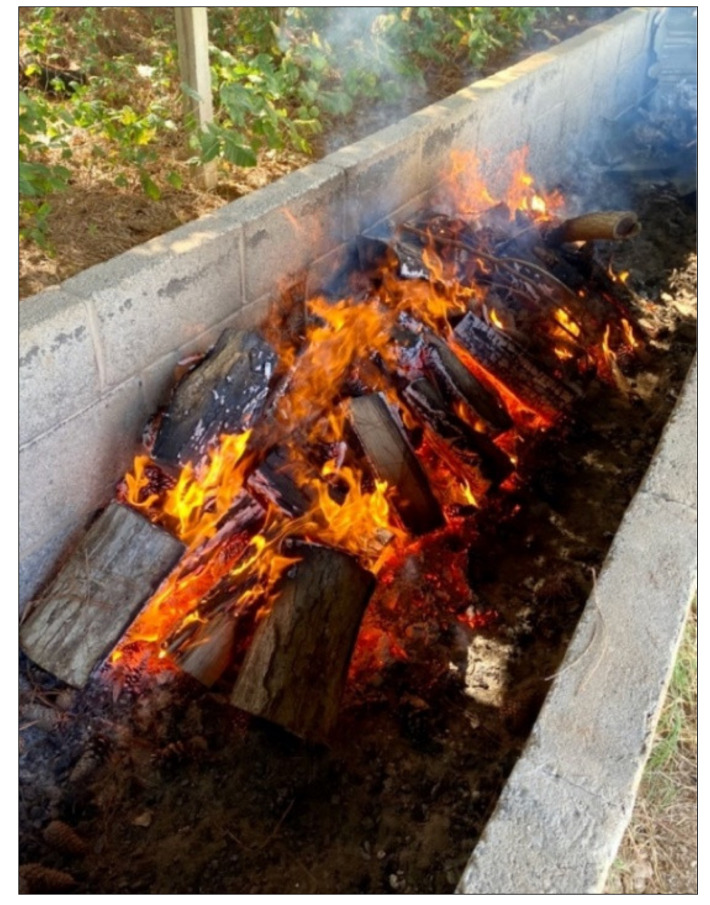
Burn site.

**Figure 3:**
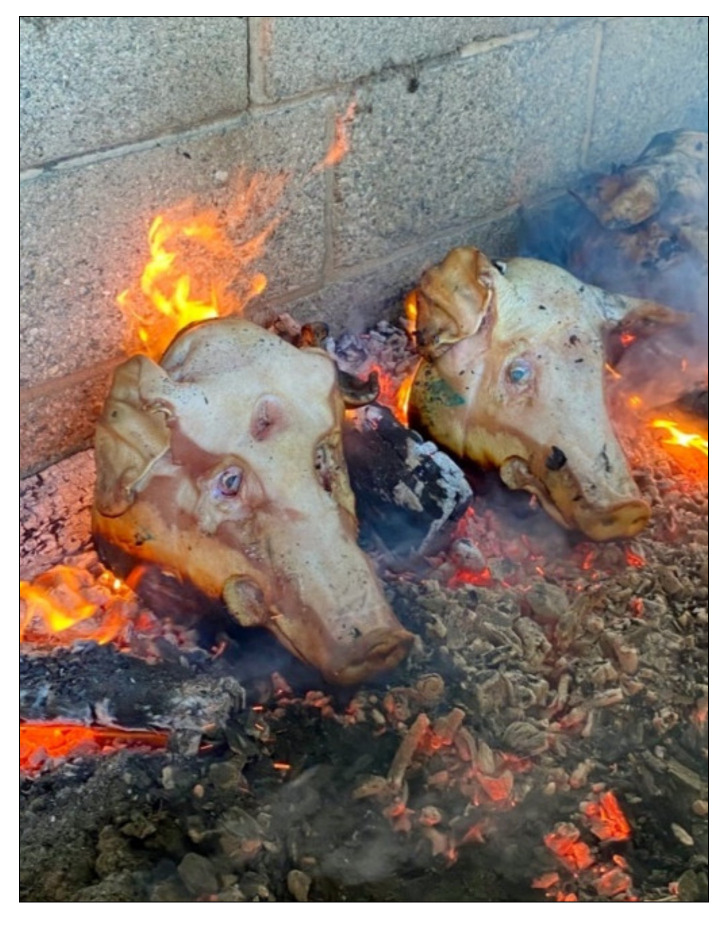
Sample placement.

The burn site was located on the North Carolina State University dairy farm and the fire was constructed within a livestock feeding trough surrounded by cinderblock walls (Figure 2). An open-air, outdoor fire was implemented for this research, as this type of deposition is commonly encountered with forensic burning scenarios [1,5,24,25,26]. Materials involved in the creation and maintenance of the fire included wood logs and coals from previous fires. No accelerants were used in the process. Each sample was positioned with the head in the horizontal plane and maintained this position for the duration of the burn cycle (Figure 3). Specimens were placed directly on top of the logs in two rows, and documentation of the progressive thermal destruction was noted via photographs during the experiment. Total burn time was 1 h and 40 min, and the samples were burned until the calcined bone was seen across the samples. Once the degree of burning was sufficient to produce largely calcinated bone, the logs were removed from the fire to slowly decrease the temperature until the samples only remained on ash. The samples were left within the fire pit overnight to allow the specimens to completely cool before removal. The following morning the skulls and associated fragments were collected by hand from the pit, placed within individual containers, and returned to the laboratory for analysis.

**Figure 4:**
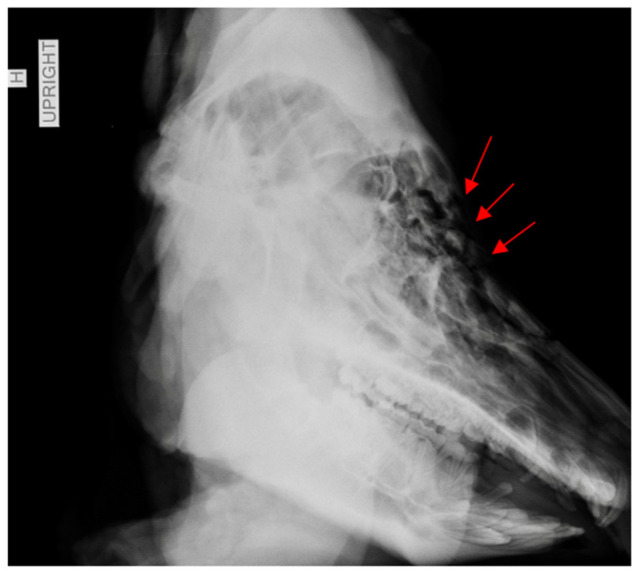
Radiographic image of specimen HH1. Red arrows point to areas of inwardly crushed bone, with displaced fragments.

**Figure 5:**
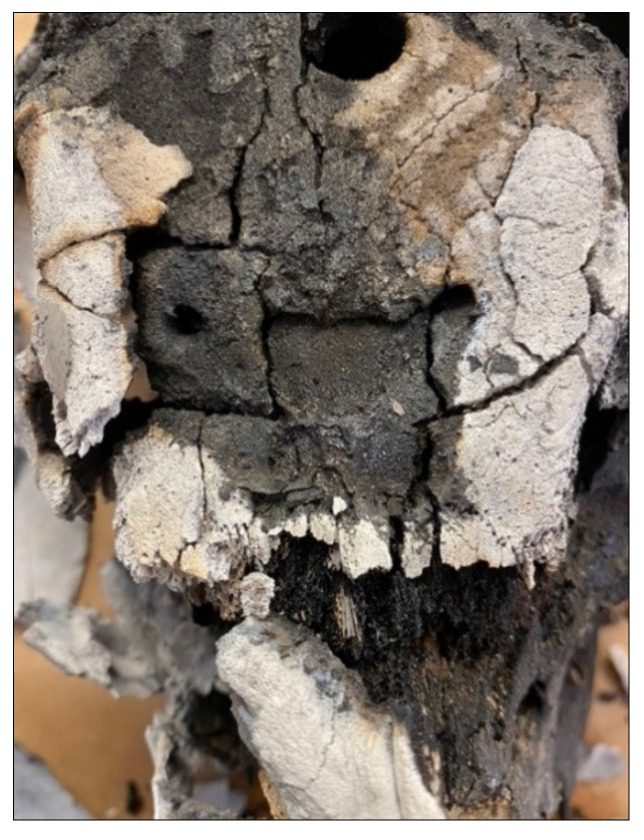
Post-processing reconstruction showing fragment association retaining depressed impact area.

Laboratory analysis began with preprocessing photographs of each skull to document differential soft tissue destruction and note any thermal signatures before soft tissue removal. Following photography, any loosely adhering soft tissue was removed with a fine, soft-bristled brush. The skulls were reconstructed by refitting fragments using an adhesive. Control samples were analyzed first so that features of thermal fractures could be noted and established before comparison with the traumatized specimens. Location of fracture origin and termination, fracture type, skeletal color changes, and areas of soft tissue survival were recorded. Traumatized samples were reconstructed in the same manner as the controls and their pre-burning radiographs were compared post-burning (Figure 4 and Figure 5).

## 3. Results

**Figure 6:**
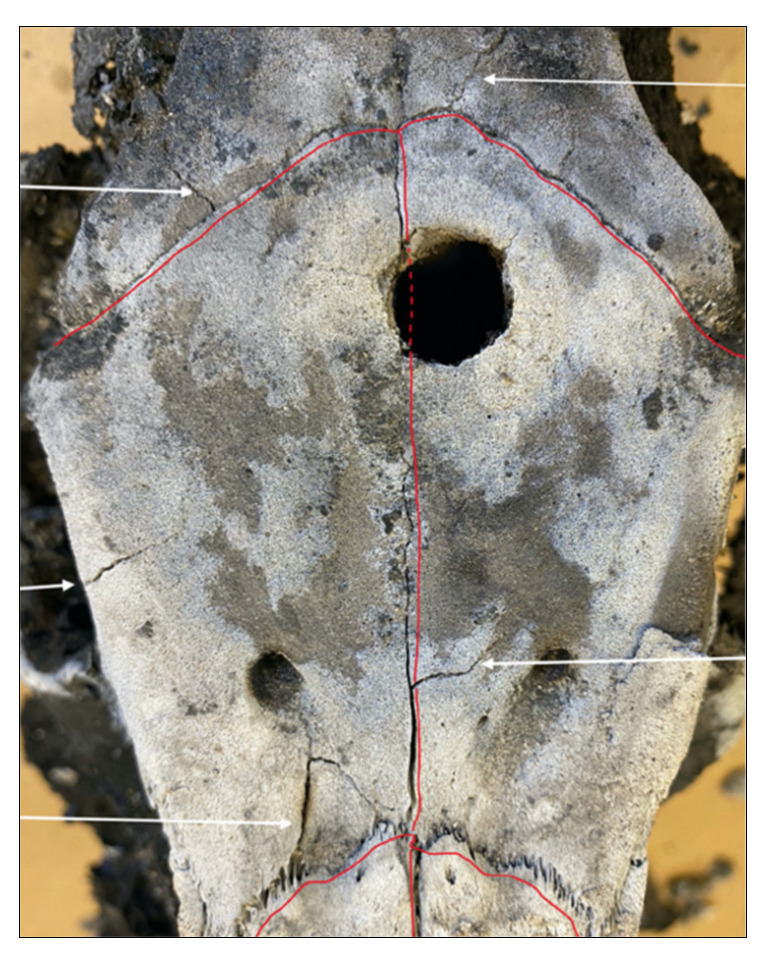
Red lines denote cranial sutures. White arrows point out thermal fractures originating and terminating within other sutures or foramina.

**Figure 7:**
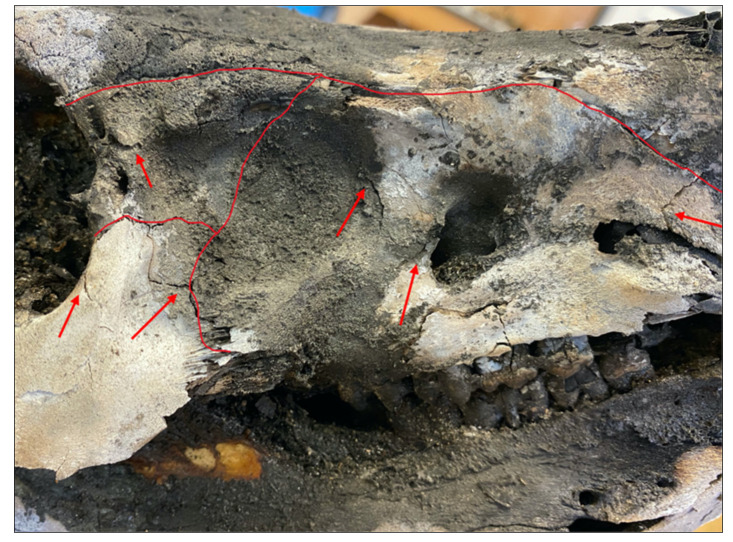
Red lines denote cranial sutures. Red arrows point out thermal fractures originating and terminating within other sutures or foramina.

All three controls showed similar thermal alterations, which consisted largely of longitudinal fractures. These longitudinal fractures were inter-connected by transverse fractures or terminated transversely into an adjacent suture. In essence, thermal fractures appeared as patterns of long, rectangular fractures all over the cranium. Further, thermal fractures were found to be associated with cranial foramina and sutures. Thermal fracture propagation consistently originated from cranial sutures or foramina and terminated into longitudinal fractures or nearby sutures Figure 6 and Figure 7. This finding was also highlighted in the study of Macoveciuc et al. (2017), who noted that due to the lack of accessory (traumatic) fractures in controls, heat accumulation caused fractures to originate from areas of the bone that could more easily vent, in this case being foramina and sutures. Thermal degradation was further characterized by cortical flaking and patina, a result of the rapid loss of organic components in the bone, and curved transverse fractures due to tissue regression [6,7,15,17,18,19,20,21].

**Figure 8:**
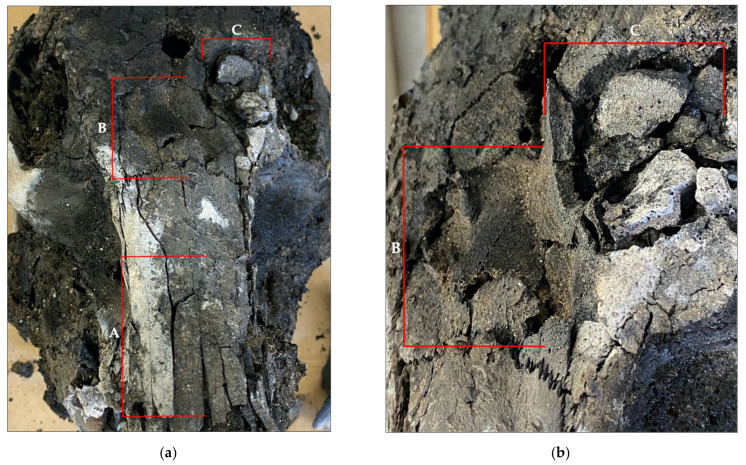
Sample CBH2 (**a**) **A** longitudinal thermal fractures interconnected with transverse fractures, **B** blunt force trauma retained showing depressed region with associated fragments, **C** comminuted fracture at impact site; (**b**) closer image of impact sites **B** and **C**.

**Figure 9:**
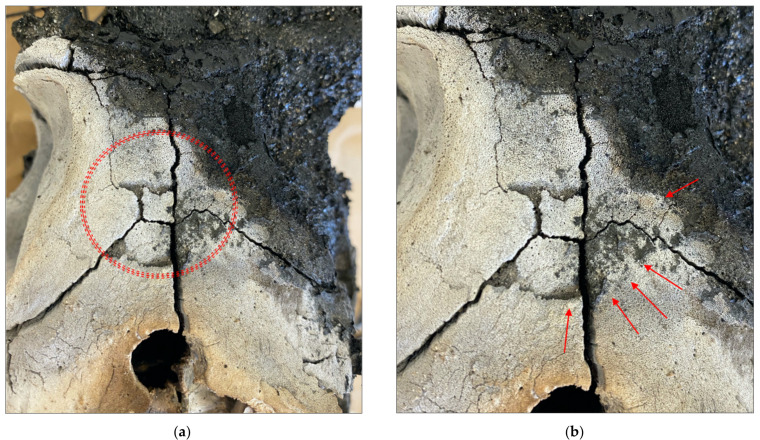
Hammer sample (**a**) region of impact exhibiting retained plastic deformation; (**b**) closer image of depression.

Traumatized samples featured distinct characteristics observed only in the specimens that underwent mechanical force, which included the post-burning retention of plastic deformation, and impact areas that featured comminuted fracturing (Figure 8). Only green (e.g., wet, living) bone can respond to force as plastic deformation, as the impacted surface absorbs compressive force causing the opposite (internal) surface to tear from tension [6,12,13,14]. In all of the samples, blunt force fractures retained the pre-burning depressed areas and, in some samples, fragments were still connected to the associated fragment through an incomplete fracture. Due to the loss of the more pliable, organic components which allow for plasticity in bone, burning bone responds as a brittle material incapable of plastic deformation [6,7,8,9]. Only traumatized regions of the crania exhibited features of depressed fractures Figure 9. When considering fracture type, blunt force impacts were almost exclusively associated with comminuted fractures, a feature absent in controls or untraumatized regions. This difference in fracture type, being primarily longitudinal or comminuted, allowed for easier identification of suspect areas of trauma. The summary of fracture type and occurrence is presented in Table 2.

## 4. Discussion

The results of this pilot study demonstrated that consistent patterns of thermal alteration were noted that allowed for the differentiation of perimortem trauma after burning. Further, we did not find that thermal alterations obscured the blunt force trauma in any of the samples. Since this study incorporated the analyses of criteria noted to be of diagnostic value in similar research [1,3,6,7], the results of these analyses are discussed further.

### 4.1. Skeletal Biomechanics: Fracture Type & Morphology

Post-burning analyses found that the structural reactions between wet and brittle/dry bone were maintained, as fracture type and morphology reflected the material state of the bone when fractured. Plastic deformation was identified as areas of inwardly crushed bone with associated fragments still partially or completely attached. Although thermal exposure altered impact areas, exhibited as patina and flaking on fractured surfaces, the areas of impact retained the general morphology of the impact (as noted on pre-burn radiographs). In cases where no plastic deformation was retained, impact areas could be identified through the reassociation of fragments using an adhesive. Reassociated fragments displayed impact areas of clustered comminuted fractures (a feature absent in the thermally altered controls). Thermally altered controls consistently exhibited longitudinal, transverse, combination longitudinal-transverse, patina, and curved transverse (i.e., from tissue regression), but did not display any areas of comminuted patterns or depressions due to plasticity. Blunt force samples also presented these thermal alterations. However, regions of trauma were easily identified as variations from these thermal characteristics. In highly fragmented specimens, where the structural integrity of the cranium is lost and plastic deformation is absent, we find that identifying fracture patterns after fragment reassociation most consistently indicated the presence of trauma. Diagnostic importance has been given to fracture type and morphology in previous studies, and this study supports that these variables are indicative of fracture cause [1,3,6,7].

### 4.2. Fracture Origin and Termination

By first assessing the controls, location patterns of thermal fracture origin and termination were established. Thermal fractures appeared to originate and terminate in areas of the skull where thermal venting was present. That is, thermal fractures could be traced to cranial foramina or sutures and terminated within adjacent foramina and sutures. This finding is consistent with other studies that conclude that the pressure of high temperatures within the cranium is released through natural vents, or openings, within the skull [1,6,7]. The pressure and heat released from this venting cause associated fractures to appear from these openings. The samples subjected to blunt force trauma showed the same fracture location patterns. However, locations of trauma deviated from this pattern as a cluster of comminuted fractures with no clear association to suture or foramina origins. Although fracture origin is variable when caused by traumatic force, thermal fractures are consistently associated with natural areas of thermal venting.

### 4.3. Skeletal Color Change

Previous suggestions [6,7] regarding color change as an indicator to differentiate fracture origin (e.g., due to thermal venting or burn progression) were not found to be useful in diagnosis for this experiment. Color changes appeared inconsistent in pattern or progression, exhibited as sporadic calcined islands surrounded by charred rings. Although potentially helpful for charting thermal degradation for remains consisting of a more complete body, these variables were not found to be of diagnostic value in this study. However, our study supports previous studies that fractures propagating into green or wet bone are associated with blunt force and perimortem trauma [1,6,7].

### 4.4. Tissue Thickness and Soft Tissue Survival

After evaluating body positioning and tissue thickness, it was observed that tissue regression and subsequent first areas of bone to burn reflected the general thickness of the tissue covering the bone, regardless of the position of the crania. The first areas to burn followed a pattern from the facial and snout regions, these being the least protected by muscle or tissue, with the thick tissues of the mandible burning last. The variability of tissue thickness across each skull created highly differential degrees of burning. Overall, facial regions were nearly calcinated while the mandible retained green bone under the lower facial muscles. Each skull consistently exhibited this pattern of tissue regression. After burning concluded, the only surviving tissues were those of the posterior portion of the mandible. Further, it appeared that fat acted as an accelerator of thermal destruction, while muscle acted as a protector. It was noted that regions of the skulls that were highly cartilaginous or fatty, such as the ears, burned more quickly than regions of the skull in more direct contact with the fire or with more densely concentrated muscle.

## 5. Conclusions

After incorporating analyses deemed to be of diagnostic value or indicative of blunt force trauma after thermal exposure in other studies, we found that the most valuable variable to identify the cause of fracture (e.g., blunt force or thermal alteration) is the fracture pattern. The result of this study found that tissue thickness is more indicative of thermal progression than body positioning and warrants further study when evaluating the progression of thermal destruction across skeletal elements.

## Figures and Tables

**Table 1:** Sample distribution. Tool column indicates classification of instrument used during manual trauma. Position lists skull pose during trauma. Identifier # lists the classification system assigned to samples throughout experimentation.

Group	Tool	Position	Identifier #
1	Control-NA	NA	C1
	Control-NA	NA	C2
	Control-NA	NA	C3
2	Crowbar	Supine	CBS1
	Crowbar	Supine	CBS2
	Crowbar	Horizontal	CBH1
	Crowbar	Horizontal	CBH2
3	Hammer	Supine	HS1
	Hammer	Supine	HS2
	Hammer	Horizontal	HH1
	Hammer	Horizontal	HH2

**Table 2:** Summary of fracture pattern observations. ✓ indicates presence of feature, X indicates absence of feature.

Sample	Defect Observed						
	Longitudinal	Transverse	Comminuted	Curved Transverse	Depressed	Patina	Delamination
C1	✓	✓	X	✓	X	✓	✓
C2	✓	✓	X	✓	X	✓	✓
C3	✓	✓	X	✓	X	✓	✓
CBS1	✓	✓	✓	✓	✓	✓	✓
CBS2	✓	✓	✓	✓	✓	✓	✓
CBH1	X	X	✓	✓	✓	✓	✓
CBH2	✓	✓	✓	✓	✓	✓	✓
HS1	✓	✓	✓	✓	✓	✓	✓
HS2	✓	✓	✓	✓	✓	✓	✓
HH1	✓	✓	✓	✓	✓	✓	✓
HH2	✓	✓	✓	✓	✓	✓	✓

## Data Availability

Images will be made available upon request.
